# Protocol to culture cells on genipin-mixed collagen gels with different stiffnesses

**DOI:** 10.1016/j.xpro.2025.104125

**Published:** 2025-10-01

**Authors:** Seiichiro Ishihara, Hisashi Haga

**Affiliations:** 1 Department of Advanced Transdisciplinary Sciences, Faculty of Advanced Life Science, Hokkaido University, N10-W8, Kita-ku, Sapporo 060-0810, Japan

**Keywords:** cell Biology, cell culture, Biotechnology and bioengineering

## Abstract

The stiffness of extracellular matrices is critical for cellular functions such as growth and differentiation. Here, we present a protocol for culturing cells on stiffness-modulated collagen gels by adding genipin, an amine crosslinker with low cytotoxicity. We describe the steps for preparing collagen gels with genipin, culturing the cells, and immunofluorescent staining of the cells on the gels. This protocol has potential applications in the analysis of function or protein/gene expression in cultured cells on extracellular matrices of different stiffnesses.

For complete details on the use and execution of this protocol, please refer to Ishihara et al.^1^

## Highlights

• Steps for culturing cells on genipin-mixed collagen gels of different stiffnesses
• Instructions for preparing genipin-mixed collagen gels with 0.0292–12.5 kPa
• Guidance for immunofluorescent staining of cells on genipin-mixed collagen gels

## Abstract (editor highlights)

Publisher’s note: Undertaking any experimental protocol requires adherence to local institutional guidelines for laboratory safety and ethics.

## Graphical abstract

**Figure:**
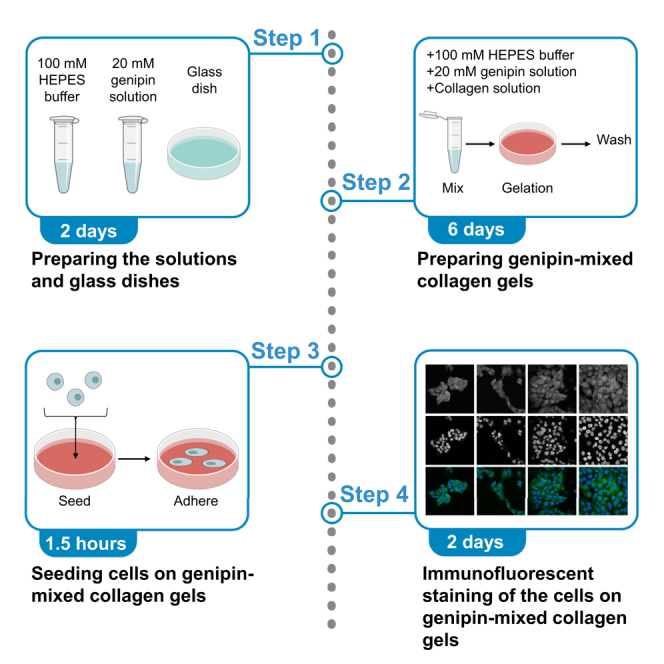

## Before you begin

The stiffness of extracellular matrices (ECMs) is critical for cellular phenomena such as growth, migration, and differentiation.^2^^,^^3^^,^^4^ Particularly, cancer cells respond to ECM stiffness and change their malignancy. For example, transcription factors such as YAP/TAZ, NF-κB, and activating transcription factor 5 (ATF5) in cancer cells are activated by stiff ECMs and promote cancer progression.^1^^,^^5^^,^^6^^,^^7^ To analyze the effects of ECM stiffness on cells, we culture cells on substrates with different stiffnesses. Collagen gels are widely used as substrates in cell cultures. The protocol below describes the specific steps for preparing collagen gels with different stiffnesses, culturing the cells on them, and immunofluorescent staining of the cells. In this protocol, we mix genipin, an amine crosslinker with low cytotoxicity, to modulate the stiffness of collagen gels.^8^ We also present a protocol for immunofluorescent staining of ATF5, a stiffness-sensitive transcription factor, in cancer cells.^1^

### Innovation

For culturing the cells on substrates with different stiffnesses, the combination of a stiff substrate (collagen-coated glass or plastic dish) and a soft substrate (collagen gel) is one of the conventional methods.^3^^,^^7^ In addition, by modulating the collagen concentration, we can change the stiffnesses of the gels, as higher collagen concentrations result in stiffer gel properties.^2^^,^^9^ These methods are convenient, however, the exact concentration of collagen in these substrates cannot be controlled. Another way to prepare substrates with different stiffnesses is using polyacrylamide gels with different crosslinker concentrations.^10^ In this way, we can coat the gels with appropriate concentrations of ECMs, however, some cells can degrade the coated ECMs, and as a result, the artificial polyacrylamide gel surface is directly exposed to the cells. Thus, a way to control the stiffness of ECMs, such as collagen gels with the same concentration of ECMs and low cytotoxicity, is needed. Therefore, we used genipin, an amine crosslinker with low cytotoxicity, to prepare collagen gels with different stiffnesses for the cell culture. We successfully modulated the stiffness of collagen gels (2.5 mg/mL collagen concentration) from 0.0292 to 12.5 kPa with low cytotoxicity.

**Figure 1:**
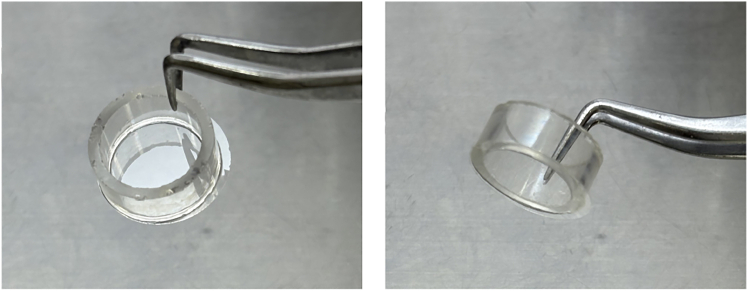
A glass dish prepared in “prepare glass dishes”

### Prepare x10 phosphate-buffered saline

**Timing: 30 min**

1. Dissolve 40 g NaCl, 5.75 g Na_2_HPO_4_, 1 g KCl, and 1 g KH_2_PO_4_ in pure water (Elix water) and complete the volume to 500 mL.

***Note:*** The solution can be stored at room temperature (20°C–25°C, it can be stored for at least 1 year).

### Prepare 100 mM HEPES buffer

**Timing: 60 min**

2. Add 2.383 g HEPES and 20 mL x10 PBS to 60 mL pure water (Elix water).
3. Adjust the pH of the solution to 7.3–7.4 by adding 1 N NaOH.
4. Make up the volume of the solution to 100 mL with pure water (Elix water).
5. Sterilize the solution with a bottle top filter.

***Note:*** The solution should be aliquoted and stored at −20°C (it can be stored for at least 1 year).

### Prepare 20 mM genipin solution

**Timing: 150 min**

6. Add 0.226 g genipin to 50 mL 100 mM HEPES buffer.

**CRITICAL:** Warm the solution with a 37°C water bath for approximately 60–120 min and vortex every 5–10 min to dissolve genipin completely.

7. Sterilize the solution with a bottle top filter.

***Note:*** The solution should be aliquoted and stored at −20°C in the dark (it can be stored for at least 1 year).

### Prepare glass dishes

**Timing: 2 days**

8. Glue a glass ring (16 mm inner diameter) to a cover glass with Cemedine Super to make a glass dish. Leave it to harden overnight (12–24 h) at room temperature (20°C–25°C).
9. Polish the glass dish surface with sodium bicarbonate. Then, wash the glass dish with tap water.
10. Clean the glass dish with ultrasound in 20% Extran MA01 in pure water (Elix water) for 10 min.
11. Wash the glass dish with running water for at least 3 h. Then, rinse it with pure water (Elix water).
12. Dry the dish at 80°C for 3 h.

***Note:*** The glass dishes can be stored at room temperature (20°C–25°C). A prepared glass dish is shown in Figure 1.

## Key resources table

**Table:** 

REAGENT or RESOURCE	SOURCE	IDENTIFIER
**Antibodies**		

Anti-ATF5, rabbit-mono (SD2099) (use at 1:200 dilution)	Novus Biologicals	Cat# NBP2-67767
Goat anti-rabbit IgG (H+L), Superclonal recombinant secondary antibody, Alexa Fluor 488 (use at 1:200 dilution)	Invitrogen	Cat# A27034

**Chemicals, peptides, and recombinant proteins**		

Dulbecco’s modified Eagle’s medium	Sigma-Aldrich	Cat# D6046
Fetal bovine serum (FBS)	Sigma-Aldrich	Cat# 172012-500ML
Antibiotic antimycotic solution (antibiotics)	Sigma-Aldrich	Cat# A5955-100ML
Atelocollagen acidic solution (collagen solution)	KOKEN	Cat# IPC-50
Genipin	Wako	Cat# 078-03021
Hoechst 33342, trihydrochloride, trihydrate	Invitrogen	Cat# H1399
Trypsin-EDTA (0.25%), phenol red (trypsin solution)	Gibco	Cat# 25200-072
NaCl	Wako	191-01665
Na_2_HPO_4_	Wako	195-05725
KCl	Wako	163-03545
KH_2_PO_4_	Wako	169-04245
HEPES	Dojindo	342-01375
Paraformaldehyde	NACALAI TESQUE	26126-54
Triton X-100	Sigma-Aldrich	30-5140-5
Skimmed milk	Megmilk Snow Brand	180 g
Sodium hydrogen carbonate	Junsei Chemical	43305-1267

**Experimental models: Cell lines**		

KP4	RIKEN Cell Bank	Cat# RCB1005; RRID: CVCL_1338

**Other**		

Confocal laser scanning microscope	Nikon	C2
Glass ring (16 mm inner diameter)	Self-made	N/A
Cover glasses	Matsunami Glass	C022001
CEMEDINE Super	CEMEDINE	CA-151
Extran MA01	Merck	1075553000

## Materials and equipment

**Table:** x1 PBS (PBS)

Reagent	Final concentration	Amount
x10 PBS	x1	50 mL
pure water (Elix water)	N/A	450 mL
**Total**	**N/A**	**500 mL**

Sterilize the solution using a bottle-top filter or autoclave. Store at room temperature (20°C–25°C, it can be stored for at least 1 year).

**Table:** DMEM (10% FBS and 1% antibiotics)

Reagent	Final concentration	Amount
DMEM	N/A	445 mL
FBS	10%	50 mL
antibiotics	1%	5 mL
**Total**	**N/A**	**500 mL**

Store at 4°C (it can be stored for at least 1 month).

**Table:** 16% paraformaldehyde

Reagent	Final concentration	Amount
paraformaldehyde	16%(v/v)	16 g
1 N NaOH	N/A	150 μL
x10 PBS	x1	10 mL
pure water (Elix water)	N/A	N/A
**Total**	**N/A**	**100 mL**

The solution should be aliquoted and stored at −20°C (it can be stored for at least 6 months). Do not repeat the freezing and thawing process.

**Table:** 4% paraformaldehyde

Reagent	Final concentration	Amount
16% paraformaldehyde	4%(v/v)	1 mL
PBS	N/A	3 mL
**Total**	**N/A**	**4 mL**

**CRITICAL:** Prepare the solution immediately before use.

**Table:** 0.5% Triton X-100

Reagent	Final concentration	Amount
Triton X-100	0.5%(v/v)	0.25 mL
PBS	N/A	49.75 mL
**Total**	**N/A**	**50 mL**

Store at room temperature (20°C–25°C, it can be stored for at least 1 year).

**Table:** 5% skimmed milk

Reagent	Final concentration	Amount
Skim milk	5%(v/v)	0.5 g
PBS	N/A	N/A
**Total**	**N/A**	**10 mL**

**CRITICAL:** Prepare the solution immediately before use.

**Table:** Primary antibody solution

Reagent	Final concentration	Amount
Anti-ATF5, Rabbit-Mono(SD2099)	1:200	1 μL
PBS	N/A	200 μL
**Total**	**N/A**	**201 μL**

**CRITICAL:** Prepare the solution immediately before use.

**Table:** Secondary antibody solution

Reagent	Final concentration	Amount
Goat anti-Rabbit IgG(H+L), Superclonal Recombinant Secondary Antibody, Alexa Fluor 488	1:200	1 μL
Hoechst 33342, Trihydrochloride, Trihydrate	1:4000	0.05 μL
PBS	N/A	200 μL
**Total**	**N/A**	**201.05 μL**

**CRITICAL:** Prepare the solution immediately before use.

## Step-by-step method details

### Preparing genipin-mixed collagen gels

**Timing: 6 days**

This section provides a step-by-step protocol for preparing genipin-mixed collagen gels with different stiffnesses for cell culture experiments.

***Note:*** All steps are performed under sterile conditions.

1. Mix 100 mM HEPES buffer and 20 mM genipin solution on ice to prepare genipin premix solution. Please see Table 1 to calculate the volume of each solution.

**CRITICAL:** Prepare the solution on ice to avoid gelation during mixing.

2. Add 500 μL of collagen solution (5 mg/mL) to the genipin premix solution and mix the solution well by gentle pipetting for 60 s on ice.

**CRITICAL:** Prepare the solution on ice to avoid gelation during mixing.

***Note:*** In this protocol, 5 mg/mL of collagen solution is used. The concentration of collagen solution may impact the stiffness of the gel.

3. Centrifuge the mixture quickly (at approximately 2000 × g for 15 sec, room temperature (20°C–25°C)) to remove bubbles.
4. Pour the mixture on an iced glass or plastic dish.

***Note:*** The volume of the mixture should be adjusted to the size of the dishes. For example, pour 220 μL of the mixture on a 16 mm inner diameter glass dish or 500 μL of the mixture into a 35 mm plastic dish.

5. Incubate the mixture-poured dish at 37°C in a humidified incubator with 5% CO_2_ for 72 h for gelation.

**CRITICAL:** Put the mixture-poured dish with water-wetted paper or another water-filled dish in a sealed plastic case to avoid drying the gels.

6. Wash the gels with PBS three times for 1 h at 37°C in a humidified incubator with 5% CO_2_.
7. Wash the gels with DMEM for 24 h at 37°C in a humidified incubator with 5% CO_2_.
8. Wash the gels with DMEM (10% FBS and 1% antibiotics) for 24 h at 37°C in a humidified incubator with 5% CO_2_.
9. The gels are then ready to be used for cell culture.

***Note:*** Prepare the gels immediately before use.

**Table 1:** Composition of the genipin-mixed collagen gels

Young’s modulus (kPa, mean value)^8^	0.0292	0.267	0.678	1.49	3.36	9.20	12.5
Genipin concentration (mM)	0	0.01	0.05	0.1	0.5	1	10
100 mM HEPES buffer (μL)	500	495.5	497.5	495	475	450	0
20 mM genipin solution (μL)	0	0.5	2.5	5	25	50	500
5 mg/mL Collagen solution (μL)	500	500	500	500	500	500	500
Total volume of mixture (μL)	1000	1000	1000	1000	1000	1000	1000

### Cell culture and seeding cells on genipin-mixed collagen gels

**Timing: 1.5 h**

This section provides a step-by-step protocol for seeding cultured cells on genipin-mixed collagen gels with different stiffnesses. As an example, this protocol shows a procedure for preparing KP4 pancreatic cancer cells on gels for immunofluorescent staining to detect ATF5 and nuclei.

***Note:*** All steps are performed under sterile conditions.

10. Culture KP4 pancreatic cancer cells on a culture dish (e.g., 6 cm dish or 10 cm dish) with DMEM (10% FBS and 1% antibiotics).
11. Prepare subconfluent KP4 pancreatic cancer cells on a culture dish (e.g., 6 cm dish or 10 cm dish).
12. Wash the cells with PBS twice.
13. Remove PBS and add trypsin solution to the dish. Incubate the cells at 37°C in a humidified incubator with 5% CO_2_ for 5 min.
14. Detach the cells with gentle pipetting and collect them in a centrifuge tube. Add equal amounts of DMEM (10% FBS and 1% antibiotics) to the tube and pipette it gently.
15. Centrifuge the tube at 161 × *g* for 2 min.
16. Remove the supernatant and add DMEM (10% FBS and 1% antibiotics) to the tube. Pipette it gently to prepare a uniform cell suspension.
17. Determine the cell density and seed 2 × 10^4^ cells in 500 μL DMEM (10% FBS and 1% antibiotics) on the genipin-mixed collagen gel in a 16 mm inner diameter glass dish.
18. Culture the cells at 37°C in a humidified incubator with 5% CO_2_ for 2 days.

### Immunofluorescent staining of the cells on genipin-mixed collagen gels

**Timing: 2 days**

This section provides a step-by-step protocol for immunofluorescent staining of ATF5 and nuclei in KP4 cells on genipin-mixed collagen gels with different stiffnesses.

19. Remove media from the dish and add 500 μL of 4% paraformaldehyde. Incubate it at room temperature (20°C–25°C) for 10 min on a shaker.

***Note:*** Perform this step in a draft chamber.

20. Wash the cells with PBS three times.

***Note:*** Perform this step in a draft chamber.

21. Add 500 μL 0.5% Triton X-100 to the dish. Incubate it at room temperature (20°C–25°C) for 10 min on a shaker.
22. Wash the cells with PBS three times.
23. Add 500 μL 5% skim milk to the dish. Incubate it at room temperature (20°C–25°C) for 30 min on a shaker.
24. Remove 5% skim milk from the dish and add 200 μL of primary antibody solution. Incubate it at 4°C overnight (12–24 h) on a shaker.
25. Wash the cells with PBS three times.
26. Add 200 μL of the secondary antibody solution (including secondary antibody and Hoechst 33342). Incubate it at room temperature (20°C–25°C) for 1 h on a shaker in the dark.
27. Wash the cells with PBS three times.
28. Add 500 μL of PBS.
29. Image ATF5 and nuclei with confocal microscopy (Nikon, C2 with x60 objective, XY dimension: 1024 pixels, 212.13 μm). Use a 405 nm laser for nuclear staining and a 488 nm laser for ATF5 staining.

**CRITICAL:** Orange and red fluorescent dyes for secondary antibody should be avoided. Genipin crosslinks emit orange and red fluorescence (excitation/emission 510–560/590 nm, and excitation/emission 590/630 nm).^11^

## Expected outcomes

**Figure 2:**
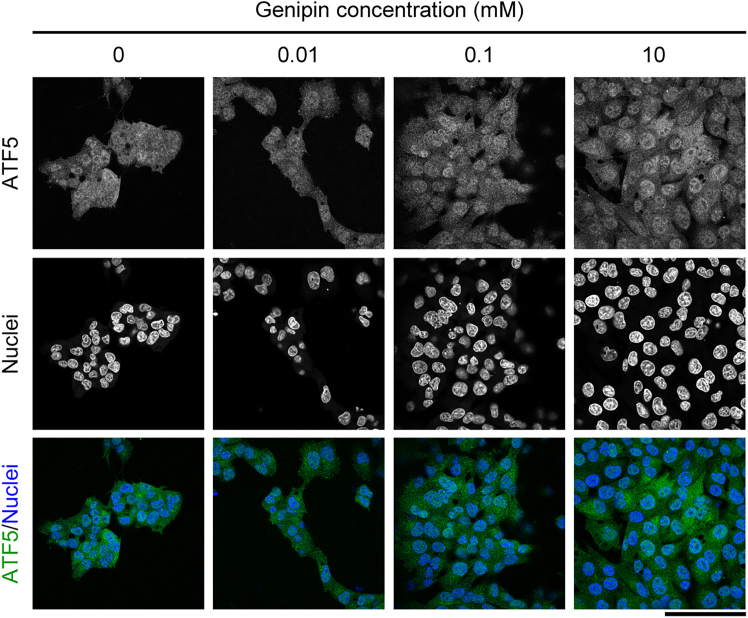
Immunofluorescent staining of ATF5 (green) and nuclei (blue) in KP4 human pancreatic cancer cells on genipin-mixed collagen gels The stiffness of gels with 0, 0.01, 0.1, and 10 mM genipin is approximately 0.0292, 0.267, 1.49, and 12.5 kPa, respectively. Scale bar = 100 μm.

Using this protocol, we can culture the cells on collagen gels of different stiffnesses and perform the following experiments. For example, specific proteins can be detected using immunofluorescent staining. We showed that ATF5 nuclear localization in KP4 human pancreatic cancer cells was promoted by stiffer collagen gels (Figure 2).^1^ These results suggested that stiff ECMs regulated ATF5 activation in pancreatic cancer cells.

## Limitations

This protocol provides stiffness-modulated collagen gels for use in cell culture experiments. However, several limitations should be noted. First, in this protocol, the maximum concentration of genipin in collagen gels is 10 mM. Therefore, the maximum stiffness of the gel is approximately 12.5 kPa. Second, we can use these gels for 2D cell culture, whereas they may not be used for 3D cell culture because free genipin in the media may affect cellular phenomena during gelation and genipin crosslinking. Third, genipin crosslinks show orange and red fluorescence potential,^11^ therefore, orange and red fluorescent dyes should not be used for fluorescent staining.

## Troubleshooting

### Problem 1

During mixing of the buffer, genipin solution, and collagen solution, the mixture starts to gel or aggregate (preparing genipin-mixed collagen gels).

### Potential solution

Keep the tube containing the solution and the mixture on ice. Warming can cause unexpected gelation or aggregation. Alternatively, chilled pipette tips can be used during the preparation of the solution.

### Problem 2

Non-uniform gels were formed after gelation in a dish (preparing genipin-mixed collagen gels).

### Potential solution

Similar to Problem 1, keep the tube containing the solution, mixture, and dishes on ice. Warming the solution and/or dishes may cause unexpected gelation or aggregation, resulting in non-uniform gels. Alternatively, use chilled pipette tips to mix the solution and pour it into dishes. In addition, centrifuge the solution in step 3 for much longer to completely remove the bubbles. Bubbles in the solution may cause non-uniform gels.

### Problem 3

Black dots appear in/on the gels (preparing genipin-mixed collagen gels).

### Potential solution

Black dots may represent aggregates of genipin with amino acids or proteins. Wash the gels more in steps 6-8. Especially, washing with PBS is critical to avoid black dots. Therefore, increasing the number of washes or the volume of PBS or media for the wash should be critical.

## Resource availability

### Lead contact

Further information and requests for resources and reagents should be directed to and will be fulfilled by the lead contact, Hisashi Haga (haga@sci.hokudai.ac.jp).

### Technical contact

Technical questions on executing this protocol should be directed to and will be answered by the technical contact, Hisashi Haga (haga@sci.hokudai.ac.jp).

### Materials availability

This study did not generate new unique reagents.

### Data and code availability

This study did not generate or analyze datasets or code.

## Acknowledgments

The authors thank Hatsumi Sano and Haruna Kurosawa for technical support and all the present and former members of Haga laboratory in Hokkaido University for helpful support. This work was supported by Advanced Research and Development Programs for Medical Innovation by 10.13039/100009619Japan Agency for Medical Research and Development (AMED) grant number JP16gm0810007 to H.H.; AMED under grant numbers JP17gm0810011 to H.H. and JP22ym0126814 to H.H.; and JSPS KAKENHI grant numbers JP17K07150 to H.H., JP21K07142 to H.H., JP18K15232 to S.I., JP21K07141 to S.I., JP23KK0143 to S.I., JP24H01917 to S.I., and JP24K10302 to S.I. This work was financially supported by Co-Creation Core for Soft Materials Aspiring Research & Translation (C3-SMART) and 10.13039/501100001691Japan Society for the Promotion of Science (JSPS) Program for Forming Japan’s Peak Research Universities (J-PEAKS) “Soft Materials Platform Aspiring the Unique Properties from Natural Polymers” and “Analysis of cancer specific mechanoresponse for drug discovery.”

## Author contributions

Conceptualization, methodology, validation, investigation, resources, writing and editing the manuscript, visualization, supervision, project administration, and funding acquisition, S.I. and H.H.

## Declaration of interests

The authors declare no competing interests.
